# Retrograde Peroneal Artery Approach to Treat Infra-Inguinal Arterial Chronic Total Occlusions: A Multicentre Experience and Technical Considerations

**DOI:** 10.3390/jcm13102770

**Published:** 2024-05-08

**Authors:** Lorenzo Patrone, Gianmarco Falcone, Raphael Coscas, Hady Lichaa, Muliadi Antaredja, Fabrizio Fanelli, Erwin Blessing

**Affiliations:** 1 West London Vascular and Interventional Centre, Northwick Park Hospital, Harrow HA1 3UJ, UK; lorenzo.patrone@nhs.net; 2 Interventional Radiology Department, Ospedale Careggi, 50134 Firenze, Italy; gianmarcofalcone@gmail.com (G.F.); fabrizio.fanelli@unifi.it (F.F.); 3 Department of Vascular Surgery, Ambroise Paré University Hospital, Assistance Publique-Hôpitaux de Paris, 92104 Boulogne-Billancourt, France; rcoscas@gmail.com; 4 Tennessee Health Science Center, Ascension Saint Thomas Heart, Nashville, TN 37203, USA; hady.lichaa@gmail.com; 5 Department of Angiology, Clinic for Vascular Medicine, University Heart and Vascular Center, University Hospital Hamburg-Eppendorf, 20251 Hamburg, Germany; m.antaredja@uke.de

**Keywords:** endovascular therapy, retrograde recanalization technique, chronic total occlusions, peripheral artery disease

## Abstract

**Background/Objectives**: Retrograde access of the peroneal artery (PA) is considered technically challenging and at risk of bleeding. The aim of this multicentre retrospective study was to assess the safety, feasibility, and technical success of this access route for infrainguinal endovascular recanalizations. **Methods**: We retrospectively analyzed 186 consecutive patients treated over a 7-year period (May 2014–August 2021) who underwent endovascular recanalization of infra-inguinal lesions using a PA access route. In all cases, retrograde PA access was obtained following a failed attempt to cross the occlusion via the antegrade route. **Results**: Among the 186 patients, 120 were males (60.5%) and the mean age was 76.8 ± 10.7 years old (44–94 years). One hundred and thirteen patients (60.7%) suffered from chronic limb threatening ischemia (CLTI). All patients presented with chronic total occlusions (CTO) and a failed conventional antegrade recanalization attempt. Retrograde access was performed under angiographic guidance in 185 cases (99.5%). It was successfully established in 171 cases (91.9%). The total rate of retrograde puncture-related complications was 2.1% (two puncture site bleedings of which one necessitated fasciotomy and two cases of arteriovenous fistulas managed conservatively). The Major Adverse Event (MAE) rate at 30 days was 1.6% (3/186). **Conclusions**: Retrograde recanalization of challenging infra-inguinal lesions via PA is safe and effective in experienced hands.

## 1. Introduction

Peripheral artery disease (PAD), particularly the more advanced stage chronic limb-threatening ischemia (CLTI), has become significantly more prevalent in the last two decades, with an estimate number of 200 million individuals affected worldwide and an amputation rate typically around 15–20% at 1 year [1,2].

CLTI is generally associated with infra inguinal lesions [3] and endovascular techniques provide benefits in terms of mortality and early complications [4]. These techniques have evolved significantly over time and are nowadays an integral part of the approach to managing patients with symptomatic PAD [5].

Endovascular recanalization of complex infra-inguinal lesions can be a challenging endeavour, with a failure rate up to 30% in the case of chronic total occlusion (CTO), if standard access techniques are used [6,7,8]. In 2005, retrograde pedal access was described for the first time to tackle complex infra inguinal disease [9] and this approach has been demonstrated to be safe and effective in several different case series [10,11,12,13,14]. The dorsalis pedis and posterior tibial arteries are considered easily accessible at the foot or the ankle level for retrograde percutaneous puncture [15], while the peroneal artery is perceived challenging to puncture and to compress due to its deep course in the leg [16].

Retrograde access of the peroneal artery was described for the first time in 2011 (ref. [17]) with the authors reporting two cases with good technical success but without subsequent limb salvage. Since then, only two main clinical papers focusing on this approach have been published [18,19], with varying success rates. In a recent systematic review [13] that included a large number of distal retrograde tibial punctures, peroneal artery (PA) access has been found to be used in only 3.8% of the all cases with the vast majority of infra-popliteal retrograde access sites obtained at the level of the dorsalis pedis artery or the distal posterior tibial artery.

The aim of this multicentre retrospective study was to assess the feasibility, technical success and safety of retrograde PA access for infra-inguinal endovascular recanalization of CTOs and to provide technical details regarding the management of this particular access route.

## 2. Materials and Methods

We retrospectively analyzed 186 consecutive patients treated by experienced operators over a 7-year period (May 2014–August 2021) who underwent endovascular recanalization of infra-inguinal CTOs using a retrograde PA access route in two high-volume centres. This study was conducted in accordance with the Declaration of Helsinki. Since the study relied on retrospective analysis of data collected during patient’s usual care, validation by an institutional review board was not necessary.

In all cases, retrograde PA access was obtained following a failed attempt to cross the occlusion via the antegrade access and the lack of a more advantageous retrograde puncture site, based on the operator’s judgement.

Technical success in obtaining PA retrograde access was defined as successfully gaining distal access followed by intraluminal wire insertion and delivery of adjunctive therapy to re-establish vessel patency with a residual stenosis of <30%. Procedural success was defined as technical success and completion of the procedure without complications. Major adverse events were defined as perioperative death (30 days) or major amputation or revascularization. A mild complication was defined as one that resolved spontaneously or with nominal intervention, did not prolong hospital stay, and did not cause permanent disability. A moderate complication included the need for significant intervention, prolongation of hospitalization for >24 h, a minor amputation, or association with minor disability that does not interfere with normal daily activity. A severe complication was defined at one that necessitated a major surgical, medical or endovascular intervention and included prolonged convalescence, major amputation, permanent disability, or death. The definitions are in line with the reporting standards of the Society for Vascular Surgery for endovascular treatment of lower extremity peripheral artery disease [20].

A 7 cm, 21 G needle was always used followed by the insertion of a 0.018guidewire (V18, Boston Scientific, Watertown, MA, USA).

Systemic heparin (starting dose equivalent to 70–80 UI/kg) was routinely administered intra-arterially to maintain the activated clotting time (ACT) between 200 and 250 s during all the procedures. In general, fluoroscopic guidance was used to perform the puncture. The needle had a retrograde 45° direction compared to the axis of the leg and was advanced in the axis of the X-rays. Once the wire advanced in the PA, crossing the lesion necessitated the insertion of a support catheter, a sheath and/or a re-entry device. The retrograde wire was then captured in the antegrade sheath to obtain a through-and-through access and complete the recanalization (see details below). Haemostasis at the level of the retrograde puncture site was successfully achieved in all cases by angioplasty with a semi-compliant balloon (2–4 mm range; size 1:1 with the angiographic diameter of the artery) from the antegrade access for a duration of 2 to 5 min and/or external compression with a blood pressure cuff. A final angiogram was always performed to confirm haemostasis at the retrograde access site prior sheath removal. The duration of postoperative antiplatelet therapy was decided according to the discretion of the operator. Generally, dual antiplatelet therapy was recommended for at least 4 weeks and up to 6 months. Over the duration of the registry, dual therapy with Rivaroxaban 2.5 mg bid and ASS 100 mg (Voyager PAD regimen) was increasingly prescribed. In case of an indication for anticoagulation, generally, a NOAC was combined with ASS for 4 weeks and monotherapy with a NOAC thereafter.

After discharge, patients were seen at the outpatient clinic with a duplex scan at 1, 6 and 12 months, and yearly thereafter. More visits were planned if needed according to the clinical situation. Continuous variables are expressed as mean ± SD with ranges. Quantitative variables are presented as numbers (n) and percentages.

## 3. Results

### 3.1. Demographics and Comorbidities

Both centres performed between 800 and 1000 endovascular procedures annually during the study period. Approximately half of those patients presented with CTOs. Retrograde recanalizations were attempted in approximately 10% of all procedures (on average, 80 per centre per year). Out of the retrograde cohort, in approximately 25% of patients, recanalization was performed via the peroneal artery or the tibioperoneal trunk.

Among the 186 patients, 120 were males (64.5%) and the mean age was 76.8 ± 10.7 years (44–94 years). The baseline characteristics of the operated patients are reported in Table 1. One hundred and thirteen patients (60.7%) suffered from CLTI. Thirteen patients (7%) presented with Rutherford class 6, seventy-eight (42%) with Rutherford class 5, twenty-two (11.8%) with Rutherford 4, seventy (37.6%) with Rutherford 3, and three (1.6%) with Rutherford 2. The target of the CTO recanalization was the femoro-popliteal segment in 75 cases (40.3%), the peroneal artery/tibio-peroneal trunk alone in 29 cases (15.6%) and a combination of these supra- and infra-genicular segments in 82 cases (44.1%).

### 3.2. Intraoperative Data

In 73 patients (39%), the antegrade access was obtained via the ipsilateral common femoral artery (CFA) and in 108 (59%) via the contralateral CFA, while in 4 (2%), the access was obtained via the brachial artery (Table 2).

Retrograde access was performed under angiographic guidance in 185 cases (99.5%) and US guided in 1 case (0.5%). It was successfully established in 171 cases (91.9%). The supplemental use of the retrograde approach allowed successful CTO lesion crossing and delivery of adjunctive therapy (including plain balloon angioplasty, drug eluting balloons, bare metal stents, drug eluting stents and atherectomy) in all the patients where the retrograde PA access was established. In all cases where CTO crossing was achieved, in line flow distal to the retrograde PA puncture site was established at the end of the procedure.

In 153 cases (89.4% of the cases where the retrograde peroneal access was successfully achieved), a sheath-less approach was used while in 18 cases (9.7%), a 4 Fr sheath was inserted to increase pushability and/or to allow introduction of larger profile catheters or re-entry devices. In 160 cases (94%), the access was used only to cross the lesion, with the definitive therapy delivered from above. In 11 cases (6%), in the interest of a low-profile approach, 6 F compatible devices, e.g., Supera stents (Abbott Vascular, Santa Clara, CA, USA) and/or an Outback re-entry device (Cordis, Miami, FL, USA), were inserted without a sheath via the retrograde PA access. Retrograde utilization of the Outback devices was used to precisely achieve re-entry into the common femoral artery in case of flush occlusion of the SFA, as previously described [21]. Retrograde deployment of the Supera stent allowed precise coverage of the origin of the SFA (PRESTO-technique) [22] when the SFA had proximal lesions. In these cases, after device removal, a 4 F sheath (which presents a 6 F outer diameter, exactly the same as the two devices mentioned above) was provisionally introduced to achieve intraprocedural haemostasis. In two cases, a 4 F sheath was intentionally inserted to safely advance a 0.018 compatible balloon from the retrograde access point to be used as a target for the puncture of an Outback device, inserted from the antegrade access, as previously described in the literature [23]. A final angiogram confirmed haemostasis at the puncture site in all cases.

### 3.3. Postoperative Results

The total retrograde puncture-related complication rate was 2.1%.

In one case (0.5%,) there was minor bleeding from a small superficial collateral, which was inadvertently hit during the puncture. Bleeding was stopped intraprocedurally with three minutes of manual external compression. In the other case (0.5%), there was postoperative bleeding at the retrograde puncture site, which required a fasciotomy due to the development of compartment syndrome. The bleeding was correlated to administration of thrombolytic therapy (10 mg of r-TPA given intraprocedural, followed by a 12 h infusion with a rate of 0.5 mg/h) because of intraprocedural formation of a thrombus at the level of the popliteal artery.

In two cases (1%), a low flow artero-venous fistula (AVF) was noticed on the completion angio at the level of the retrograde puncture site with no further treatment required (Table 2).

The 30-day amputation free survival rate was 98.9%, while the 6-month amputation free survival (calculated on 123 pts—8 died; 55 were lost to follow up) was 98.3% and the 12-month major amputation free survival was 86.3% (calculated on 88 pts—98 died or were lost to FUP). The Major Adverse Event (MAE) rate at 30 days was 1.6% (3/186), all of which were not procedural-related deaths.

## 4. Discussion

The retrograde approach to endovascular recanalization of infra-inguinal lesions was first described more than 30 years ago [24] and since then, there have been multiple reports in the literature with various retrograde access techniques described [8,9,10,11,12]. Technical procedural success is of utmost importance especially in patients with CLTI because delay or failure to appropriately improve the arterial supply in a timely fashion could result in a significant deterioration of the affected limb and ultimately in major amputation [25]. In selected cases, retrograde access via the peroneal artery can be the only chance to achieve lesion crossing and in-line arterial blood supply to the foot.

In most of our cases, retrograde access at the level of the PA was attempted to perform recanalization of a single vessel run-off peroneal artery itself or of the tibioperoneal trunk. Several authors reported similar results in terms of limb salvage and ulcer healing between revascularization procedures using PA or pedal collaterals and those obtained through direct revascularization of the appropriate angiographic angiosome [26,27]. This stresses the importance of establishing in line arterial flow in the PA, when this represents the single BTK vessel run off, with more and more recognition achieved during the last few decades for its role in achieving limb salvage [28,29].

At the same time, even when the target of the recanalization is above the knee level, the peroneal artery is the infra-popliteal vessel, which is anatomically in line with the femoro-popliteal axis. Because of its anatomical position, technically speaking, it represents the most ideal conduit to recanalize supra-genicular CTOs, providing better pushability and wire torquability during lesion crossing when compared to pedal vessels, which usually present a lower calibre and longer distance from the supra-genicular target and curve(s) between the puncture site and the target lesion.

On the other hand, historically, the main concerns regarding retrograde puncture of the peroneal artery are due to its anatomic deep location, which is more challenging in terms of access, and the potentially higher risk of bleeding due to challenging external compression [13,30]. Regarding the bleeding risk, Mustapha et al. described 23 cases of retrograde puncture of the PA and reported 7.4% cases of PA pseudo aneurysm [31]. They correlated these complications with an abrupt sheath size step-up to be added to the impossibility of achieving direct haemostasis due to the lack of antegrade access using their technique (tibiopedal arterial minimally invasive retrograde revascularization—TAMI), which expects all the treatment tools to be inserted from single retrograde infra-popliteal access via a 5–6 Fr thin-walled sheath.

In our series, we also observed significantly delayed bleeding, which was correlated with the intraoperative administration of the recombinant tissue plasminogen activator (r-tPA). We experienced a similar problem in another patient (outside from this case series) where the proximal anterior tibial artery was used as a retrograde access site and again, r-tPA was administered. In these cases, when thrombolytic drugs need to be administered intra-arterially during the procedure, we do believe that no material other than the needle and the support catheter should be used from the retrograde access point to avoid creating a larger puncture hole. Deployment of a covered stent at the level of the retrograde puncture site could represent a bail-out option in order to prevent delayed and poorly controllable bleeding. The other complication correlated with retrograde peroneal access described in the literature is the creation of the AV fistula between the peroneal artery and the peroneal vein at the site of the puncture [32], where the authors of the paper decided to place a covered stent in order to stop the significant shunt into the venous system. Placing a covered stent in a tibial vessel may cause the vessel to be at risk of thrombosis or loosing collaterals. In our cases series, we had two cases of angiographically detected AV fistulas at the site of the puncture. In both cases, it was felt that the shunt was not significantly affecting the distal flow and no covered stent was deployed at that level.

In this series, we did not experience any significant spasms or thrombosis once the retrograde peroneal access was achieved despite the lack of retrograde injection of intra-arterial vasodilators as previously described [31]. In order to minimize the thrombosis risk, we consider that it is mandatory to achieve an ACT value between 200 and 250 s, with an initial bolus of 80 unit/kg followed by maintenance doses depending on the length of the procedure and follow-up ACT values.

**Figure 1:**
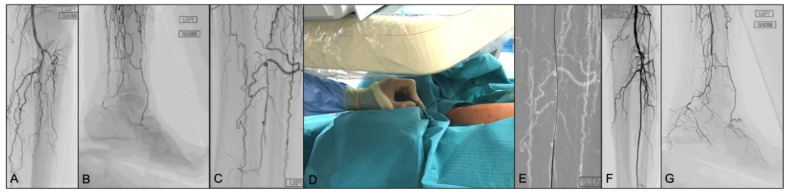
Rutherford 6 CLTI patient with no patent arterial vessel reaching the left forefoot. Peroneal artery is occluded few centimetres below its origin with distal reconstitution of the vessel just above its anterior and posterior branches division (**A**,**B**). After a failed antegrade recanalization attempt, the distal peroneal artery was visualized under fluoroscopic guidance (**C**) in the 25-degree left anterior oblique (LAO) position to visualize the artery between the fibula and tibia. A 21-gauge needle (7 cm) was advanced with an angle of approximately 45 degrees (**D**), aligned straight with the vessel smart-mask. A 0.018 wire was introduced through the needle, once bleeding was controlled (**E**). The final result after intraluminal recanalization subsequent angioplasty, with direct flow reaching the forefoot via the reconstituted peroneal artery (**F**,**G**).

**Figure 2:**
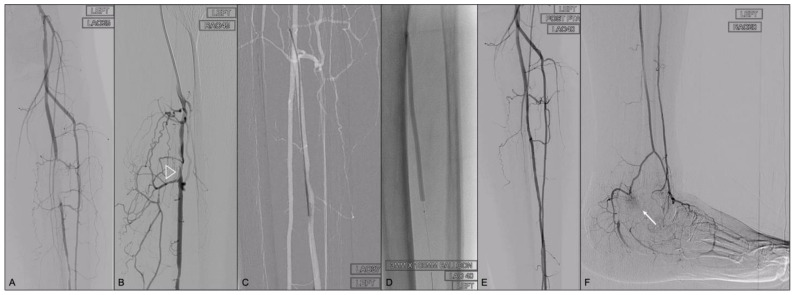
Rutherford 5 CLTI patient with non-healing left lateral malleolar ulcer. Peroneal artery occluded at its proximal-mid segment (**A**). Antegrade subintimal recanalization failed because the re-entry was achieved at the level of a PA branch (white arrow head) (**B**). The distal peroneal artery was visualized under fluoroscopic guidance (**C**) at the 37-degree left anterior oblique (LAO) position. A 23-gauge needle (7 cm) was advanced and a 0.018 wire was passed through the needle once bleeding was controlled (**C**). The final result after intraluminal recanalization and subsequent angioplasty (**D**), with direct flow reaching the ulcer and angiographic blush demonstrated (white arrow) (**E**,**F**).

With regards to the retrograde peroneal puncture, we used the distal popliteal/tibio-peroneal trunk puncture technique, using fluoroscopic guidance, independently described by Silvestro et al. [33] and Tan et al. [34] in 2018. However, we would like to focus on the following key points (illustrated in Figure 1 and Figure 2), which are specifically important for peroneal artery punctures:

- Due to its deep location, the retrograde puncture of the PA under US guidance, as described by Mustapha et al. [31], could be challenging and complete fluoroscopic guidance via the lateral compartment was preferred in our case series.
- A gentle medial rotation of the leg is usually helpful to open the gap between the tibia and the fibula without requiring excessive obliquity of the C arm.
- The following parallax principle must be outlined: the needle should be aligned with the long axis of the road-mapped vessel. The PA can be punctured at almost any level apart from the very distal tract where the needle cannot reach the target due to bone interposition. To use an angiographic subtracted image as guidance for the puncture is preferrable when compared with fluoroscopic guidance by arterial calcifications to minimize access in calcified/more diseased segments of the PA. The use of nerve block, spinal or general anaesthesia could help in terms of movement artifact reduction.
- We found that in the vast majority of our cases, two different angiograms in two orthogonal planes and contrast injections when the needle is close to puncturing the artery, as described by Zhuang [17], are not necessary. If the needle is kept in line with the course of the PA, as assessed under fluoroscopic guidance, it will impact the target artery approximately 4–5 cm from the skin. The tactile feeling generated by the passage through the interosseous membrane means that the artery is roughly 1 cm away. We think that our simplified technique could save at least 20 mls of contrast compared to the mentioned Zhuang approach [17].
- We consider a mini-invasive sheath-less approach, using a 0.018 compatible catheter (CXI, COOK MEDICAL, Bloomington, Indiana or Navicross, Terumo, Tokyo, Japan) and a 0.018 guidewire with a stainless steel shaft (V18, Boston Scientific, Marlborough, MA or Command 018, Abbott Vascular, Santa Clara, CA, USA) as a safer approach than retrograde sheath placement [35].

The main reasons for failure in achieving a stable retrograde peroneal access were the short distance between the puncture site and the level of the CTO, causing a lack of support, and the puncture-related dissection of the vessel, with the guidewire not being able to advance intraluminally.

If the target vessel for revascularization is the PA/TPT, we suggest accessing the PA at least 4–5 cm below the distal cap of the CTO. This distance usually allows the operator to have enough stability and support to effectively engage the vascular occlusion with a guidewire and microcatheter. This means that the access on the skin needs to be at roughly 7–8 cm from the diseased segment. Another tip could be to use a larger and shorter needle first to stabilize the 21 G needle progression in the soft tissues before entering the artery. In such cases, a longer 21 G needle could be necessary [36].

Despite good primary technical success and low complication rates with retrograde techniques, their use is nowadays almost always determined by the failure to cross the target lesion in an antegrade fashion, and more than likely after multiple attempts and damage to the distal landing zone [35]. As such, undertaking a robust comparison between the long-term patency of the vessels recanalized using a single antegrade versus a combined antegrade/retrograde or single retrograde approach would only be possible in a randomized controlled fashion, since failure of antegrade wire passage is likely to be associated with a higher complexity level in terms of lesion and patient characteristics. Despite this limitation and the relatively poor follow-up rates, compatible with a retrospective case series between different centres, our data show a reasonable medium-term amputation free survival rate after retrograde recanalizations using the peroneal artery as an access site.

Given the high technical success and reasonably low complication rates, we believe that, in experienced hands, retrograde access via the PA artery does not have to be reserved for CLTI patients but could also be offered to patients affected by lifestyle limiting claudication. Recently, easier retrograde access to the PA has been described [37]. It consists of puncturing under ultrasound guidance a distal termination branch of the PA that has a superficial course anterior to the lateral malleolus (mainly the anterior lateral malleolar artery). Although the precise rate of patients with a favorable configuration for this puncture site has not yet been determined, it could represent an interesting alternative to the conventional percutaneous PA access used in the present paper. In summary, we consider peroneal access particularly advantageous in (a) cases when the peroneal artery or the tibio-peroneal trunk itself are occluded and (b) in cases of very complex femoropopliteal occlusions, when pushability and wire torquability are key to procedural success and we advise against that access site in the case of very small calibers and/or presence of heavy calcification.

Regarding the limitations of this study, due to the retrospective design of our registry, we had a rather high number of patients lost to follow up. Long-term clinical outcome data (wound healing and amputation-free survival in CLTI patients and improvement of Rutherford category and in pain-free walking distance in claudicants) are clearly needed to further support the concept of the liberal use of retrograde recanalizations via the peroneal artery or the tibio-peroneal trunk. Our present registry focused rather on feasability, acute technical success and peri-procedural safety of that increasingly used access site.

## 5. Conclusions

Retrograde recanalization of challenging infra-inguinal arterial CTO via peroneal access is safe and effective. Since the procedures were performed by experienced operators in high-volume centres, larger studies are needed to confirm the safety and effectiveness of that access site. Also, long-term follow-up data including access-site-related complications need to be generated before this technique can be applied more broadly.

## Figures and Tables

**Table 1:** Baseline characteristics of the included patients.

Variable	(n = 186)
Age (years)	76.8 ± 10.7
Sex (male/female)	120 males, 66 females
Diabetes	73 (39%)
Hyperlipoproteinaemia	87 (49%)
Hypertension	48 (26%)
Coronary artery disease	71 (38%)
Patient presentation	
Rutherford class 2	3/186 (1.6%)
Rutherford class 3	70/186 (37.6%)
Rutherford class 4	22/186 (11%)
Rutherford class 5	78/186 (42%)
Rutherford class 6	13/186 (7%)
Target recanalization vessel	
Femoro-pop tract	75/186 (40.3%)
Peroneal/TP trunk	29/186 (15.6%)
ATK vessels and PA	82/186 (44.1%)

TP: tibioperoneal; ATK: above the knee; PA: peroneal artery.

**Table 2:** Procedural data.

Variable	(n = 186)
**Proximal approach**	
Ipsilateral CFA	73 (39%)
Contralateral CFA	108 (59%)
Brachial	4 (2%)
Retrograde PA/TP trunk	186 (13/186 tibeoperoneal trunk)
Access success	91.9% (171/186)
Intraluminal GW delivery failure	15/186 (7.5%)
**Procedure variables**	
Use of 4 F sheath	18/186 (9.7%)
6 F profile device-inserted retrograde	11/186 (6%)
**Vascular-access-related complications**	
Peroneal access bleeding (minor)	1 (0.5%)
Low flow A-V fistula	2 (1%)
Delayed peroneal bleeding	1 (0.5%)
Emergency amputation	0
MAE (not related to vascular access)	3 pts died during periprocedural time (1.6%)

CFA: common femoral artery; PA: peroneal artery; TP: tibioperoneal trunk; GW: guide wire; A-V: arteriovenous; MAE: major adverse event.

## Data Availability

The datasets are not publicly available.
